# Integrated Analysis of LncRNA-mRNA Coexpression in the Extracellular Matrix of Developing Deciduous Teeth in Miniature Pigs

**DOI:** 10.1155/2019/6159490

**Published:** 2019-01-23

**Authors:** Yang Li, Guoqing Li, Fu Wang, Xiaoshan Wu, Zhifang Wu, Jinsong Wang, Chunmei Zhang, Junqi He, Hao Wang, Songlin Wang

**Affiliations:** ^1^Molecular Laboratory for Gene Therapy and Tooth Regeneration, Beijing Key Laboratory of Tooth Regeneration and Function Reconstruction, School of Stomatology, Capital Medical University, Beijing 100050, China; ^2^Department of Stomatology, Beijing Tian Tan Hospital, Capital Medical University, Beijing 100050, China; ^3^Department of Oral Basic Science, School of Stomatology, Dalian Medical University, Liaoning 116044, China; ^4^Department of Biochemistry and Molecular Biology, Capital Medical University School of Basic Medical Sciences, Beijing 100069, China

## Abstract

Miniature pigs, a valuable alternative model for understanding human tooth development, have deciduous teeth from all four tooth families that are replaced once by permanent molars. The extracellular matrix (ECM) supports cells and maintains the integrity of tooth germs during tooth development. However, details on the role of the ECM in tooth development are poorly understood. Here, we performed long noncoding RNA (lncRNA) and messenger RNA (mRNA) expression profiles in the ECM components of deciduous tooth germs by RNA sequencing in miniature pigs. From the early cap to the late bell stages, we identified 4,562 and 3,238 differentially expressed genes (DEGs) from E40 to E50 and E50 to E60, respectively. In addition, a total of 1,464 differentially expressed lncRNAs from E40 to E50 and 969 differentially expressed lncRNAs from E50 to E60 were obtained. Kyoto Encyclopedia of Genes and Genomes (KEGG) pathway analysis showed that DEGs were enriched significantly for multiple signaling pathways, especially for the ECM pathway. We then outlined the detailed dynamic gene expression profiling of ECM components during deciduous molar development. Comparison of the cap and bell stages revealed that the structure and functions of the ECM dynamically changed. The ECM-related genes, including* THBS1, COL4A5, COL4A6, COL1A1, CHAD, TNR, GP1BA*, and* ITGA3*, were significantly changed, and some were shown to enrich during the bell stage development. Finally, we outlined the coexpression of lncRNAs and ECM properties during tooth development. We showed that the interplay of key lncRNAs could change ECM processes and influence the ECM establishment of tooth patterns to accomplish full tooth formation. These results might provide information to elucidate the regulation network of the lncRNA and ECM in tooth development.

## 1. Introduction

Tooth maturation is supported by alterations at the cellular and molecular levels, including gene regulation, which underlies all biological behavior and phenotypes [1, 2]. Current understanding of the molecular mechanisms controlling tooth development is mostly obtained from studies on mice, which have continuously growing incisors, three molars in each jaw quadrant, and a diastema region between the incisor and the molars, lacking canines, premolars, and secondary tooth dentition [3]. The miniature pig (Sus scrofa) resembles humans in its anatomy, physiology, pathophysiology, and development, providing an excellent experimental model for studying tooth development [4, 5].

Over the years, patterns of gene expression have been studied in developing teeth in numerous laboratories around the world [6]. A microarray study showed that about 4,000 differentially expressed genes (DEGs) were related to murine tooth development by comparing tooth germs at different time points [7]. Many studies have also revealed a wide range of functional activities of long noncoding RNAs (lncRNAs), including chromatin remodeling, transcriptional control, and posttranscriptional processing [8, 9]. The regulation of lncRNAs might contribute to odontogenesis, while the dysregulation of lncRNAs is associated with the loss of odontogenic potential [10].

Previously, we reported the developing morphology and chronology of diphyodont dentition in miniature pigs [4]. We have outlined gene expression profiling and identified 1,542 DEGs using microarrays during early deciduous molar morphogenesis in miniature pigs [11]. RNA-Seq can provide much more valuable insights into the gene expression levels, differential expression, broader range of detection sensitivity, alternative splice forms, and novel transcripts, which are not easily obtained by microarrays [12]. Outlining the complete gene and lncRNA expression profiling during tooth morphogenesis in miniature pigs is required.

The extracellular matrix (ECM) is a stationary skeleton that not only supports cell movement and the maintenance of tooth germ integrity, but also plays a role in the biological guidance and homeostasis of cells during tooth development [13]. The complicated remodeling of the ECM during tooth development draws great research interests, involving cell-cell interaction, cell-ECM interaction, molecular signaling, and pattern formation [14]. However, little is known about the role of the ECM in tooth development. Along with cell division, the ECM is produced and laid down around cells. The ECM bears the tension from the cells and tissues surrounding it, stores molecules, and provides pathways for cells and molecules for survival, migration, proliferation, differentiation, and so on [15]. To date, an in-depth analysis of the tooth germ ECM has not been reported. The present study explained the nature of ECM remodeling from the cap stage to the late bell stage, as well as a novel understanding of the relationship between lncRNAs and ECM assembly during tooth development, in miniature pigs. Dental tissue engineering efforts have yet to identify scaffolds that instruct the formation of bioengineered teeth of a predetermined size and shape [16]. Here, we investigated the ECM molecules present in natural tooth germs and how they can provide insight into how to achieve biomimetic scaffolds for dental tissue engineering.

## 2. Materials and Methods

### 2.1. Ethics Statement

Experiments were performed in strict accordance with the recommendations of the Administration of Affairs Concerning Experimental Animals (Ministry of Science and Technology, China). All procedures involving animals were approved by the Animal Care and Use Committee of Capital Medical University, Beijing, China (Permit Number: AEEI-2015-095). In brief, pregnant miniature pigs at embryonic days 40, 50 and 60 (E40, E50, and E60) were anesthetized with a combination of 6 mg/kg ketamine chloride and 0.6 mg/kg xylazine. After removing the fetuses by cesarean section, the pregnant sows were sacrificed by overanesthetization. [17]

### 2.2. Tissue Acquisition and Total RNA Isolation

We chose E40, E50, and E60 stage corresponding to the cap stage, early bell stage, and late bell stage mandibular third deciduous molar (Dm3), respectively. The Dm3s were harvested from the staged embryo mandible under a stereomicroscope (LEICA), rinsed in sterile phosphate buffered saline (PBS), placed into 150 *μ*L RNAlater stabilization solution (ThermoFisher Scientific) for 16 h at 4°C, and then stored at −20°C. Total RNA from Dm3 at each stage was extracted separately using a miniKit (QIAGEN, Germany) according to the manufacturer's protocol. RNA purity was checked using the NanoPhotometer spectrophotometer (IMPLEN, USA). Total RNA was then quantified on a NanoDrop ND-2000 (Thermo Scientific, USA). The RNA integrity was assessed using an Agilent Bioanalyzer 2100 (Agilent Technologies, USA), and the RNA with RIN (RNA integrity number) > 8.0 is acceptable for transcriptome library construction. [17, 18]

### 2.3. Transcriptome Sequencing

The RNA sequencing libraries were generated using the rRNA-depleted RNA by Directional RNA Library Prep Kit for Illumina (NEB, USA). First strand cDNA was synthesized using random hexamer primer and M-MuLV Reverse Transcriptase (RNase H). Second strand cDNA synthesis was subsequently performed using DNA Polymerase I and RNase H. PCR was performed with Phusion High-Fidelity DNA polymerase, universal PCR primers and Index (X) Primer. The PCR products were purified (AMPure XP system) and library quality was assessed on the Agilent Bioanalyzer 2100 system. The detailed method was used as previously described [19].

The clustering of the index-coded samples was performed on a cBot Cluster Generation System using HiSeq 2500 PE Cluster Kit (Illumina). After cluster generation, the library preparations were sequenced on an Illumina Hiseq 2500 platform and 125 bp paired-end reads were generated. The transcriptome sequencing was performed by NovelBio Corp. Laboratory, Shanghai, China. Raw data were firstly processed through in-house perl scripts to obtain clean data by removing reads containing adapter, ploy-N and with low quality that reads with >5% ambiguous bases (noted as N) and low-quality reads containing more than 20 percent of bases with qualities of <13. At the same time, Q20, Q30, and GC content of the clean data were calculated to meet the standard (Q20>90, Q30>85) [20].

### 2.4. Reads Mapping and Data Analysis

All the downstream analyses were based on the clean data with high quality. The clean reads were aligned to the reference genome (Sus scrofa from UCSC) using TopHat v2.0.9 [17, 21, 22]. Differential expression analysis between biological replicates per condition was performed using the DESeq R package (1.10.1) [23]. The resulting P-values were adjusted using the Benjamini and Hochberg's approach for controlling the false discovery rate (FDR). Expression levels of the transcripts, including lncRNAs and mRNAs, were quantified as fragments per kilobase of exon per million fragments mapped (FPKM). Differential gene expression was determined using Cuffdiff with a FC (fold change) ≥ 2 and P-value <0.05 and FDR <0.05 between the different developing stages at E40, E50 and E60. [24]

### 2.5. Gene Set Enrichment Analysis

The functions of DEGs were chosen for Gene Ontology (GO) analysis with online tool (DAVID, http://david.abcc.ncifcrf.gov/). Pathway analysis was used to place differentially expressed coding genes according to Kyoto Encyclopedia of Genes and Genomes (KEGG). Generally, Fisher's exact test followed by Benjamini–Hochberg (BH) multiple testing correction was calculated to select the GO category and the significant pathway. A p value less than 0.05 is considered to be statistically significant.

### 2.6. PPI Network Analysis and Gene Coexpression Networks Analysis

Protein-protein interaction (PPI) network analysis of DEGs was based on the Search Tool for the Retrieval of Interacting Genes/Proteins (STRING) database, which contains known and predicted PPIs. We constructed the PPI networks by extracting the target gene lists of the ECM pathway from the database.

We created coexpression networks to track the interactions between lncRNAs and DEGs. Gene coexpression networks were created according to the normalized expression values of genes from ECM pathway-related search terms. For each pair of genes, we calculated the Pearson correlation and choose the significant correlation pairs (false discovery rate [FDR] < 0.05) to construct the network. Accordingly, the genes with the highest degrees of connection were identified as the key regulatory genes and lncRNAs. Network structure analysis was used to locate the core regulatory factors (genes). While considering different networks, the core regulatory factors were determined by the degree of difference between the two class samples. The network was visualized using Cytoscape software (version 3.6.1).

## 3. Results

### 3.1. Global Gene Expression Profiles Specific to Pig Tooth Development

The developmental morphology of tooth germs at key development stages was shown in Supplementary Figure 1. Generally, the characteristics of the tooth germ at E40 are the cap-shaped enamel, the dental papilla, and the dental sac enclosing them; compared to E40, the cap-shaped enamel organ was transformed into a bell-shaped structure at E50, in which differentiation occurred to its furthest extent in the cells forming the inner enamel epithelium, stratum intermedium, stellate reticulum, and outer enamel epithelium; the enamel organ at E60 reached the late bell stage, in which the proteins and organic matrix form a partially mineralized enamel and dentin.

**Figure 1:**
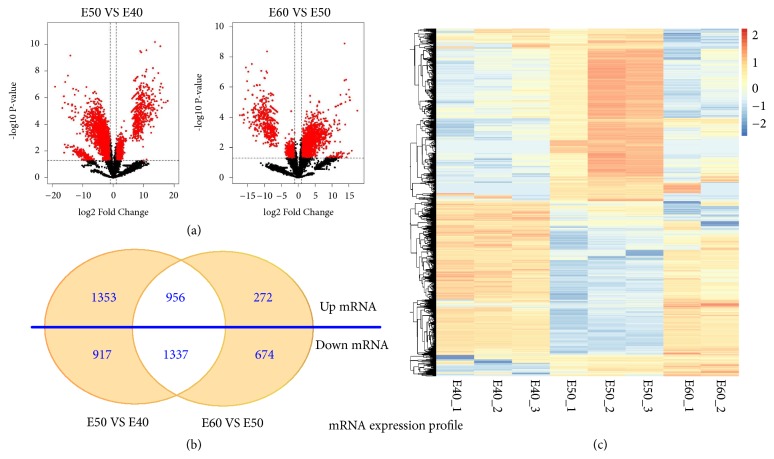
**mRNA expression profile at the E40, E50, and E60 stages of deciduous tooth germs.** (a) Volcano plots of mRNA expression levels between E40 and E50 (left) and E50 and E60 (right). (b) Venn diagram of upregulated and downregulated mRNAs from E40 to E50 (left circle), from E50 to E60 (right circle), and the merged part of differentially expressed mRNAs (*P* < 0.05; fold change > 2.0). (c) Heatmap of differentially expressed mRNAs. Red represents upregulated genes while blue represents downregulated genes.

**Figure 2:**
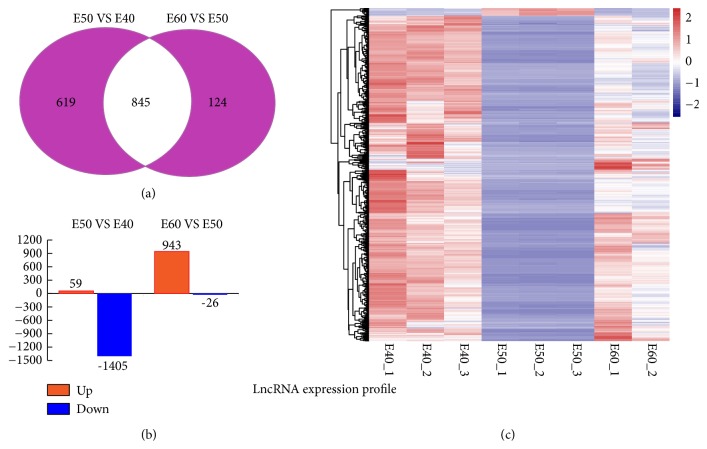
**lncRNA expression profile at E40, E50, and E60 stages of deciduous tooth germ.** (a) Venn diagram of differently expressed lncRNAs during E40 to E50 and E50 to E60 stages. (b) Histogram of lncRNA expression levels from E40 to E50 (left), from E50 to E60 (right). (c) Heatmap of the differently expressed lncRNAs at E40, E50, and E60. The small panel with color gradient represents the changes in abundance from downregulated (blue) to upregulated (red) genes.

We analyzed the mRNA and lncRNA expression levels measured in the tooth germs E40, E50, and E60. The sequence data is deposited in Gene Expression Omnibus (GEO) of National Center for Biotechnology Information (NCBI) database with GEO accession code GSE122516. We found that the gene expression patterns showed significantly different expression levels among the E40, E50, and E60 stages. The volcano plot in Figure 1(a) was created to identify differences among mRNAs by setting stringent criteria using the EBSeq algorithm (fold changes [FC] > 2 or FC < 0.5 and FDR < 0.05). The Venn diagram and clustered heatmaps of DEGs are shown in Figures 1(b) and 1(c). The DEGs were identified during the transition from the cap- to bell stage (E40 to E50); 3,238 DEGs were identified, including 1,599 upregulated genes, and 1,639 downregulated genes (Supplementary Table 1). During the transition from the early bell to late bell stage (E50 to E60), 4,562 DEGs were identified, including 2,309 upregulated and 2,253 downregulated genes (Supplementary Table 2). We identified 1,464 differentially expressed lncRNAs during the transition from the cap to bell stage (E40 to E50), including 59 upregulated genes, and 1,405 downregulated lncRNAs (Supplementary Table 3). We also identified 969 differentially expressed lncRNAs during the transition from the early bell to late bell stage (E50 to E60), including 943 upregulated genes, and 26 downregulated lncRNAs (Supplementary Table 4). The Venn diagram, histogram, and clustered heatmaps of differentially expressed lncRNAs are shown in Figures 2(a), 2(b), and 2(c). As an initial framework for understanding lncRNA function, lncRNAs can be broadly classified into those that act in* cis*, influencing the expression and/or chromatin state of nearby genes and those that execute an array of functions throughout the cell in* trans* [25, 26]. Interestingly, the majority of the expressed novel lncRNAs were downregulated during the cap stage, but upregulated during the bell stage in our data set. The potential mechanism for this remains to be further explored.

### 3.2. Tooth Development-Related Genes Are Enriched in ECM Pathway

**Figure 3:**
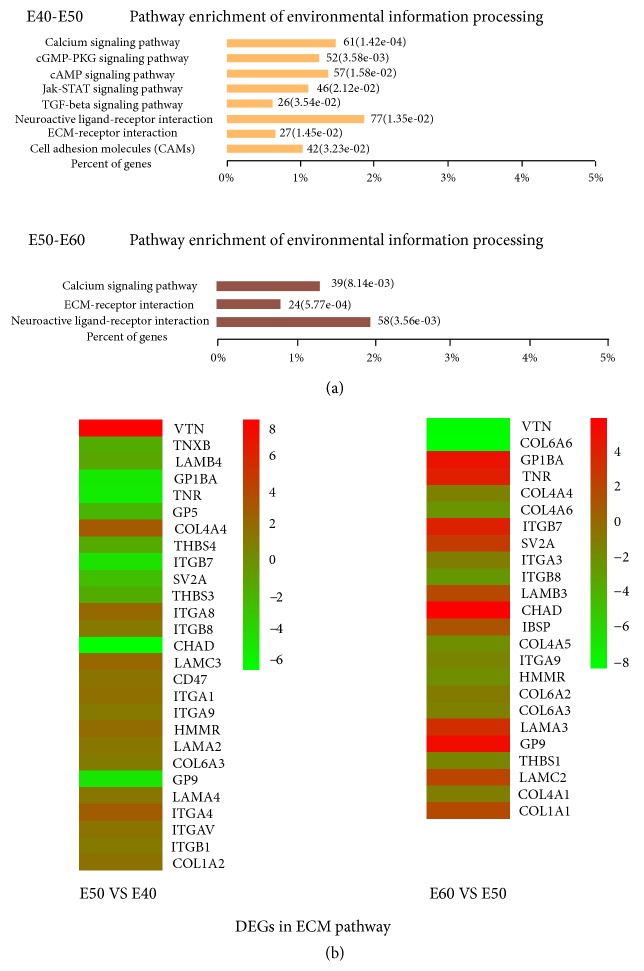
**KEGG pathway and significant functional genes of the stage-dependent ECM receptor interaction pathway.** (a) KEGG pathways were enriched for the environmental information processing category. The number of genes in the pathway search term and* P*-value were at the right end of the horizontal rectangle. The horizontal axis was the percentage of genes in the pathway search term and total number of genes for a selected pathway search term. (b) Heatmap showing genes of the ECM pathway based on the relative abundance (log⁡2[fold change ratios]). The small panel with color gradient represents the changes in abundance from downregulated (green) to upregulated (red) genes.

The significantly enriched KEGG pathways were identified (*P* < 0.05, FDR < 0.05) (Supplementary Table 5). The functional pathway analyses demonstrated potential modulating pathways, such as ECM receptor interaction pathways, the TGF-*β* signaling pathway, and the calcium signaling pathway. We focused on the ECM receptor interaction pathways, which play a key role in environmental information processing (Figure 3(a)).

The tooth consists of three principal mineralized tissues: enamel, dentin, and the surrounding bone. Development of these tissues requires ECMs that contain distinct sets of genes. We obtained global and novel ECM gene expression profiles during the cap and bell stages. The expression changes in the ECM pathway genes from the cap to the bell stage are shown in Figure 3(b) and Supplementary Table 6. Some of the ECM genes first increased, then decreased, such as* VTN, COL4A4, ITGA8*, and* ITGA4*; some decreased continually, such as* COL6A3*; some first downregulated then upregulated such as* CHAD, TNR,* and* GP9*. During tooth development, the collagen genes, such as* COL4A4, COL4A6, COL6A3, COL1A1*, and* COL6A6*, changed dramatically.* COL4A4* was the main collagen component during the cap stage.* COL1A1* increased sharply at the time of early bell stage and considered to play the dominant role in the mechanical support.* ITGB7, LAMB3, LAMA3*, and* LAMC2* were upregulated markedly, suggesting the important roles in the ECM pathway during the bell stage. Basement membranes (BMs) are the first extracellular matrices to appear in development [27]. They not only provide the scaffold for cells and cell layers, but also play an essential role in morphogenesis, which affects cell adhesion, migration, proliferation, and differentiation. BMs have been reported to consist of* COL4, LAMA,* and other molecules that were detected in our research and interact with each other to form supramolecular structures [28]. The structures and components of ECMs were tooth-specific structures and functions. Further analysis showed there were simultaneous bindings of the miRNA to the candidate lncRNA and mRNA (Figure 5, Supplementary Table 7), such as, LOC102165412 targeting THBS1 by ssc-miR-7143-5P. The colocalization of the candidate lncRNAs and mRNAs (Supplementary Table 8), such as LOC102164680 colocated with COL1A1 and LOC102165704 coexpressed with COL1A (Supplementary Figure 2).

### 3.3. The Protein-Protein Interaction in the ECM Pathway

**Figure 4:**
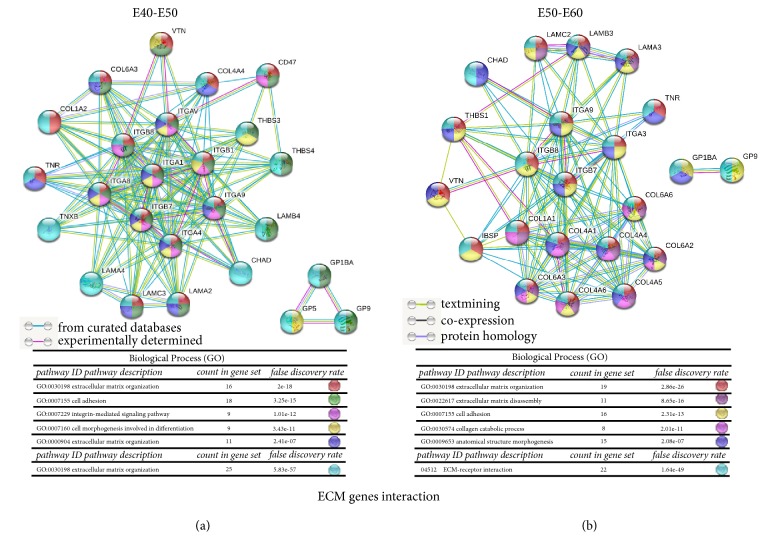
**Gene interactions of ECM components of developing deciduous teeth.** (a) Interaction of genes involved in the ECM pathway from E40 to E50. (b) Interaction of genes involved in the ECM pathway from E50 to E60. The local clustering coefficient > 0.7. Line color indicates the type of interaction evidence. The DEGs of ECM pathway were colored according to the biological process. The GO ID of each gene, pathway description, count in gene set, and false discovery rate of every gene in the ECM pathway were noted in the sheets.

We subjected the ECM DEG profiles of tooth development to the STRING database (https://string-db.org) and focused on gene-gene interaction in the ECM pathway during E40 to E50 (Figure 4(a)) and E50 to E60 (Figure 4(b)) using a high confidence score (coefficient > 0.7). The ECM DEGs were* ITGA, ITGB, COL1, COL4, COL6, THBS, LAMA, LAMB, LAMC*, and* GP*. In this network,* THBS1, COL1A1, COL4A1, COL4A6, LAMB3, LAMC2, *and* LAMA3* have experimentally determined interactions. Gene ontology (GO) analysis (http://geneontology.org/), which is the key functional classification in the National Center for Biotechnology Information (NCBI) database (http://www.ncbi.nlm.nih.gov/), was used to determine the main function of the ECM genes. During the cap stage (E40 to E50) the ECM DEGs were involved in most aspects of cellular processes, including development, differentiation, growth, and apoptosis. The GO enrichments were extracellular matrix organization, cell adhesion, integrin-mediated signaling pathway, cell-matrix adhesion, and cell morphogenesis involved in differentiation. However, during the bell stage (E50 to E60), the ECM DEGs were enriched in terms of their extracellular matrix organization and disassembly, collagen catabolic processes, and anatomical structure morphogenesis.

### 3.4. The lncRNAs Closely Associated with the ECM Pathway

**Figure 5:**
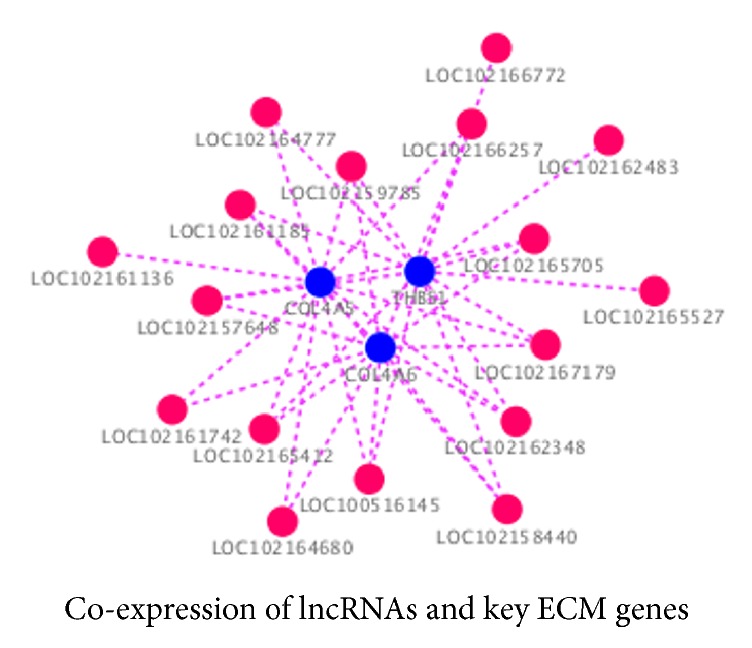
**LncRNA-mRNA coexpression network of* COL4A5*,* COL4A6*, and* THBS1* in the ECM pathway.** An lncRNA-mRNA coexpression network was constructed based on the Pearson correlation coefficients. Pink color nodes represent lncRNA, while blue nodes represent the coexpression genes. Dotted lines represent the correlation.

To further identify the relationship between the ECM pathway and lncRNAs, a diagram of the lncRNA modulation of the ECM signaling pathway was created to provide useful clues on the mechanism associated with tooth development. Within the pathway, 39 unique ECM genes were enriched. Additionally, joint analysis showed 845 lncRNAs were coexpressed with ECM genes (Supplementary Figure 2).* COL4A6, COL4A5*, and* THBS1* were coexpressed with the lncRNAs LOC102165705, LOC102164777, LOC100516145, etc. The genes, including* TNR, SV2A, ITGB7, ITGB1, ITGB8, VTN, GP5, GP9, THBS3, ITGA3, ITGA9, GP1BA, LAMA2, LAMA4, LAMC3*, and* CHAD*, encode essential signaling molecules for the ECM pathway, and the coexpression networks indicated correlations between all lncRNAs and mRNAs in the ECM pathway (Figure 5).

## 4. Discussion

In the present study, we identified 4,562 and 3,238 DEGs from the E40 to E50 and E50 to E60 tooth germs, respectively. We also obtained a total of 1,464 and 969 differentially expressed lncRNAs from E40 to E50 and from E50 to E60, respectively. The DEGs were subjected to KEGG pathway analysis, and were found to be significantly enriched for the ECM receptor interaction pathway. ECM genes, such as* LAMA3, COL1A1, CHAD, TNR, GP1BA*, and* ITGB7*, were significantly upregulated and were shown to contribute specifically to the bell stage development, but the ECM genes* THBS1, COL4A4, COL4A1*, and* COL4A6* were significantly downregulated during the bell stage. Previous studies showed the interactions among* THBS1, COL1A1, COL4A1*, and* COL4A6, *which are regulated by lncRNAs; therefore, these interactions may change ECM processes, especially the collagen components, for accomplishing full tooth formation.

The ECM is a fundamental component of the microenvironment of cells and has been substantially expanded during the evolution of vertebrates [13]. Some of this expansion has contributed to structural components, but it is evident that this is only one role of the ECM. The ECM provides much more than mechanical support and a locus for cell adhesion, and native ECMs are necessary for cellular recognition and interaction, in addition to overcoming various technical challenges in regulating tooth sizes and achieving high porosity structures [29]. Dental tissue engineering efforts have yet to identify scaffolds that instruct the formation of bioengineered teeth of predetermined size and shape [30]. Here, we identified novel key genes involved in the ECM during deciduous molar development, so that we also can investigate whether ECM molecules present in natural tooth scaffolds can provide insights into achieving tooth regeneration.

Transcriptional regulation is fundamental to understanding the molecular mechanism of tooth development [31, 32]. However, at present, none of the known lncRNAs have demonstrated functional roles in tooth development in large mammals [33]. The basic goal of this study was to establish a database of lncRNA expressions during tooth formation, which could provide a broad foundation to guide focused studies of lncRNA function and regulation of mechanisms that govern the maturation of tooth germ processing. RNA-Seq is the method of choice for gene expression analysis due to the accuracy and the wide dynamic range of the data generated [9]. This extensive RNA-Seq comparison allowed, for the first time, a gene interaction analysis of ECM genes during the cap and late bell stages of tooth development. Here, we also generated complete lncRNAs transcriptome profiles in miniature pigs with a high throughput RNA-Seq method. The differential analyses of global lncRNA expression revealed significant differences between E40, E50, and E60 germs. Our previous study showed that from the early stage of the cap (E40) to the early bell stage (E50), the mRNA expression pattern of miniature pigs was downregulated by a large number of genes From E50 to E 60, the mRNA expression pattern was upregulated by a large number of genes [11]. The regulation of mRNA is similar to the regulation of lncRNA. It is considered that the period from the cap stage to the bell stage is the key stage of tooth crown formation, and there are a large number of cell differentiation changes from E40-E60. The results also revealed previously unknown tooth germ ECM components, including* COL4A5, COL4A6, THBS1,* and key regulation lncRNAs, that were found to contribute to ECM remodeling.

By showing that genes and lncRNAs have functions together, this study identifies a deciduous molar core developmental program in large mammalian species. The data served as indicators for the subsequent wide search for “key regulatory genes and lncRNAs” in tooth development. The expression of classical activation markers that have been reported previously may account for the tooth regeneration process, though further investigations are required to clarify the underlying mechanisms during tooth morphogenesis.

## 5. Conclusions

By comparing the mRNA and lncRNA expressions at the key developmental stages of deciduous tooth germ development by RNA-Seq, we identified 4,562 and 3,238 DEGs from E40 to E50 and E50 to E60, respectively. In total, 1,464 and 969 differentially expressed lncRNAs from E40 to E50 and E50 to E60 were obtained, respectively. Many of these mRNAs were not detected in a previous microarray-based experiment, suggesting that distinct platforms detect different sets of DEGs. The lncRNA profiles of deciduous tooth germs were established for the first time. Ten significantly enriched pathways from all DEGs were partially consistent with previous reports. Using RNA-Seq, we have provided the first transproteomic characterization of the tooth ECM remodeling process in miniature pigs. We found that the cooperation between 845 lncRNAs induces the activation of ECM signaling at the bell stage. The interplay of key lncRNAs could change ECM processes by their interaction with key genes. Matrix components that, in turn, signal the appropriately timed translation and secretion of ECM components result in a well-ordered matrix. Stage-specific ECM remodeling lays the foundation for the future use of biomimetic scaffolds for dental tissue engineering applications.

## Data Availability

The data used to support the findings of this study are available from the corresponding author upon request.

## Supplementary Materials

Supplementary Materials Supplementary Table 1: the identified differentially expressed genes during the deciduous tooth germ transition from the cap stage to bell stage (E40 to E50) (XLS 100 kb). Supplementary Table 2: the identified differentially expressed genes during the deciduous tooth germ transition from the early bell stage to late bell stage (E50 to E60) (XLS 71kb). Supplementary Table 3: the identified differentially expressed lncRNAs during the deciduous tooth germ transition from the cap stage to the early bell stage (E40 to E50) (XLS 211kb). Supplementary Table 4: the identified differentially expressed lncRNAs during the deciduous tooth germ transition from the early bell stage to the late bell stage (E50 to E60) (XLS 626 kb). Supplementary Table 5: the significantly enriched KEGG pathways associated with the grouped DEGs (XLS 169kb). Supplementary Table 6: the expression changes of the ECM pathway genes from E40 to E50 and from E50 to E60 (XLS 11kb). Supplementary Table 7: the miRNA-target lncRNA and mRNA regulatory interactions of the ECM pathway (XLS 27kb). Supplementary Table 8: lncRNA and mRNA colocation analysis of the ECM pathway genes (XLS 30kb). Supplementary Figures: the developmental morphology of tooth germs at key development stages E40, E50, and E60 (Supplementary Figure 1). The coexpressed analysis of ECM genes and lncRNAs (Supplementary Figure 2) (DOCX 15mb).
