# Supplementary material for: Integrated Analysis of LncRNA-mRNA Coexpression in the Extracellular Matrix of Developing Deciduous Teeth in Miniature Pigs
Source: Biomed Res Int. 2019 Jan 23;2019:6159490. doi: 10.1155/2019/6159490 (PMC6364112; doi:10.1155/2019/6159490)
Supplement: Supplementary Materials — Supplementary Table 1: the identified differentially expressed genes during the deciduous tooth germ transition from the cap stage to bell stage (E40 to E50) (XLS 100 kb). Supplementary Table 2: the identified differentially expressed genes during the deciduous tooth germ transition from the early bell stage to late bell stage (E50 to E60) (XLS 71kb). Supplementary Table 3: the identified differentially expressed lncRNAs during the deciduous tooth germ transition from the cap stage to the early bell stage (E40 to E50) (XLS 211kb). Supplementary Table 4: the identified differentially expressed lncRNAs during the deciduous tooth germ transition from the early bell stage to the late bell stage (E50 to E60) (XLS 626 kb). Supplementary Table 5: the significantly enriched KEGG pathways associated with the grouped DEGs (XLS 169kb). Supplementary Table 6: the expression changes of the ECM pathway genes from E40 to E50 and from E50 to E60 (XLS 11kb). Supplementary Table 7: the miRNA-target lncRNA and mRNA regulatory interactions of the ECM pathway (XLS 27kb). Supplementary Table 8: lncRNA and mRNA colocation analysis of the ECM pathway genes (XLS 30kb). Supplementary Figures: the developmental morphology of tooth germs at key development stages E40, E50, and E60 (Supplementary Figure 1). The coexpressed analysis of ECM genes and lncRNAs (Supplementary Figure 2) (DOCX 15mb). [file 6159490.f1.zip › Supplementary materials/Supplementary Figures.docx]

Supplementary materials


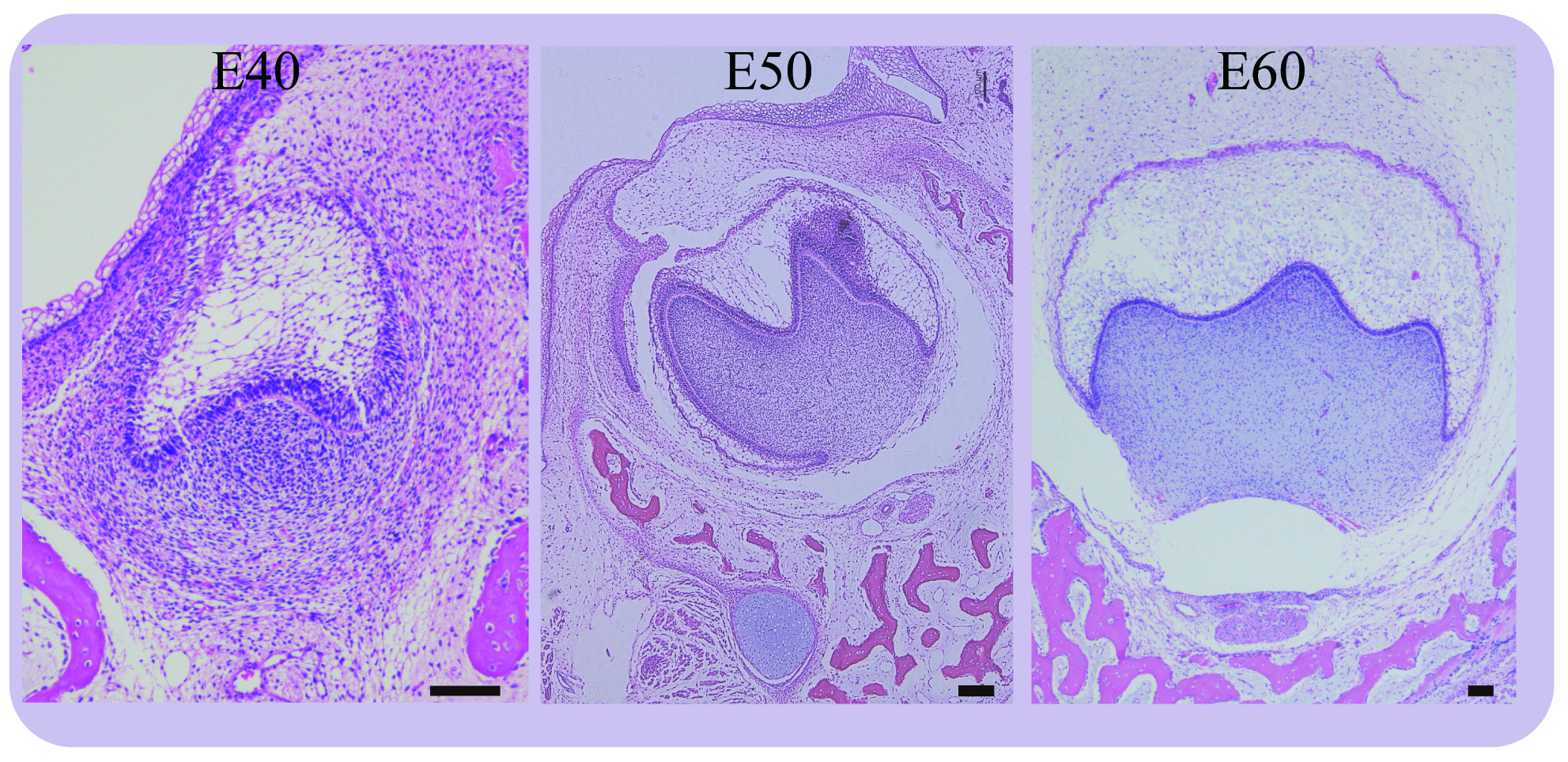


**Supplementary Figure** 1. **Developing patterns of the third deciduous molar (Dm3) from the mandible in the miniature pig model.**

Coronal histological sections (hematoxylin and eosin staining) show that the E40 Dm3 in the cap stage, E50 Dm3 in the early-bell stage, and E60 Dm3 in the late-bell stage (secretory stage). E: embryonic day. Scale bar = 100 um.


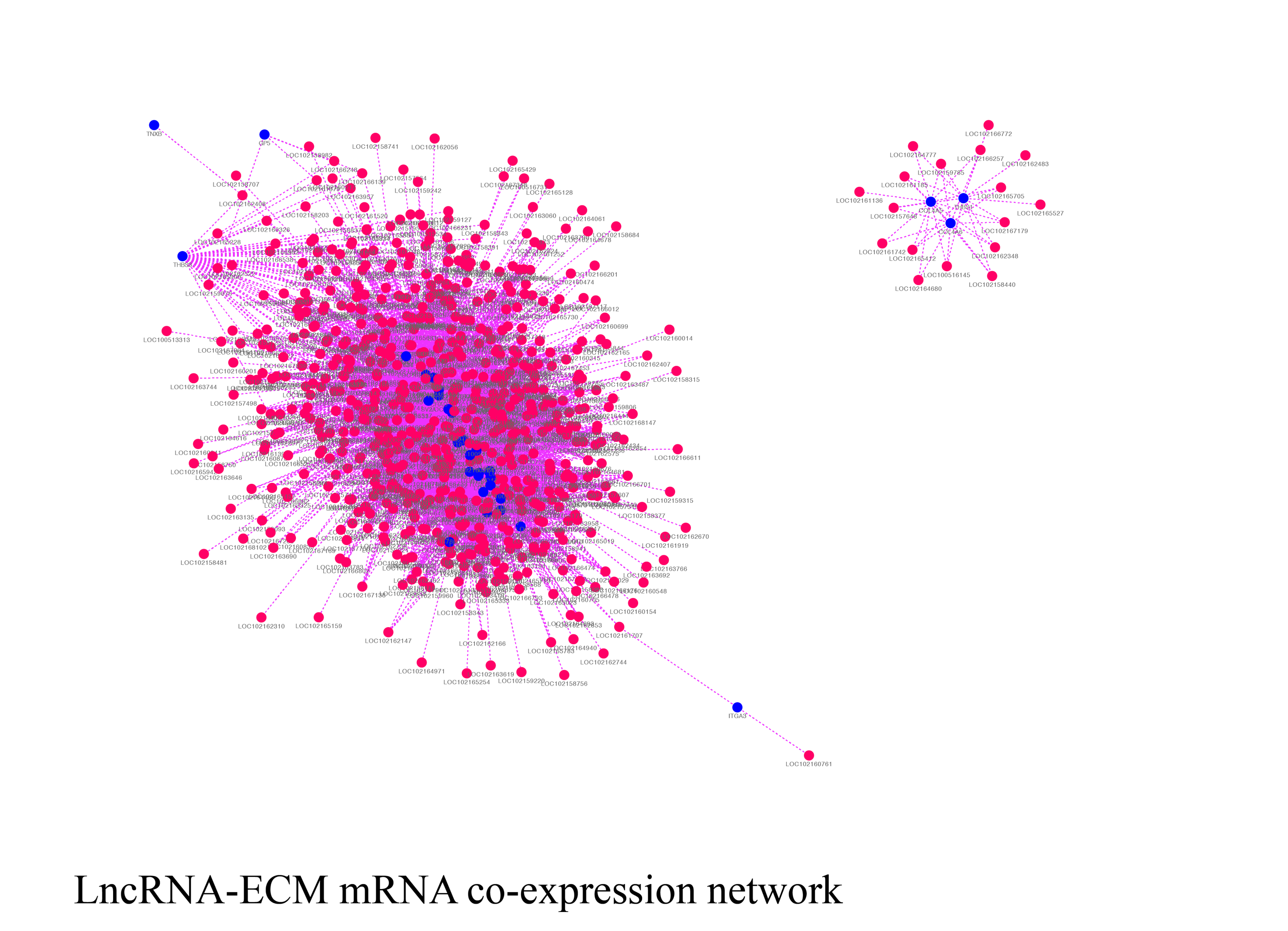


**Supplementary Figure 2. LncRNA-mRNA co-expression network in ECM pathway.**

The lncRNA-mRNA co-expression network was constructed based on the Pearson correlation coefficients. Pink color nodes represent lncRNAs, while blue nodes represent co-expression genes. Dotted lines represent the correlation.
